# Clinical and Genetic Characteristics of *IKZF1* Mutation in Chinese Children With B-Cell Acute Lymphoblastic Leukemia

**DOI:** 10.3389/fgene.2022.822832

**Published:** 2022-03-28

**Authors:** Jingying Zhang, Xiao-Jun Xu, Lixia Liu, Hua Song, Heping Shen, Weiqun Xu, Fenying Zhao, Juan Liang, Chan Liao, Yan Wang, Tian Xia, Shanbo Cao, Yongmin Tang, Jiayue Qin, Diying Shen

**Affiliations:** ^1^ Division/Center of Pediatric Hematology-Oncology, The Children's Hospital of Zhejiang University School of Medicine, Hangzhou, China; ^2^ The Pediatric Leukemia Diagnostic and Therapeutic Technology Research Center of Zhejiang Province, Hangzhou, China; ^3^ National Clinical Research Center for Child Health, Hangzhou, China; ^4^ Acornmed Biotechnology Co., Ltd., Tianjin, China

**Keywords:** IKZF1 mutation, B-cell acute lymphoblastic leukemia, genetic characteristics, clinical features, targeted next-generation sequencing

## Abstract

Acute lymphoblastic leukemia (ALL) is a malignancy associated with altered lymphoid precursor hyperplasia and accompanied with different genetic mutations. Few studies have been reported on the association between gene mutations and clinical features of *IKZF1* mutation in children with B-cell ALL (B-ALL). We investigated clinical and genetic characteristics in 200 newly diagnosed pediatric B-ALL through multiplex ligation-dependent probe amplification (MLPA) and targeted next-generation sequencing (NGS) method. We found that *IKZF1* mutations, including large segment deletions, small insertions or deletions (InDels) and single nucleotide variations (SNVs), were detected in 22 patients with a positive mutation rate of 11.0%. *IKZF1* mutation was significantly associated with higher WBC count (19.38 × 10^9^/L vs. 5.80 × 10^9^/L, *p* = 0.002). Compared with *IKZF1* wild-type cases, a higher frequency of *IL7R* gene mutation was discovered in *IKZF1* mutant cases (9.1% vs. 0.0%, *p* = 0.012). Patients with *IKZF1* mutation were less sensitive to glucocorticoid induction than patients without *IKZF1* mutation (63.6% vs. 9.0%, *p* < 0.001). On the 15th day of induction, minimal residual disease (MRD) > 10^−3^ level were higher in *IKZF1* mutant patients than wild-type patients (45.5% vs. 22.3%, *p* = 0.018). In conclusion, our study reveals the association between genetic mutations and clinical features in Chinese children with B-ALL, which might contribute to molecular classification, risk stratification and prognosis evaluation, and provide new ideas for targeted therapy in ALL.

## Introduction

Acute lymphoblastic leukemia (ALL) is a malignancy associated with altered lymphoid precursor hyperplasia, and about 75% of children with ALL develop chromosomal changes, such as aneuploidy, translocation, copy number changes, or gene rearrangements (Holmfeldt et al., 2013). With the wide development of genome-wide analysis, some ALL children have *IKZF1* mutation, including large segment deletion, small insertions or deletions (InDels) and single nucleotide variations (SNVs), which is considered to be a marker of poor prognosis in pediatric ALL.

*IKZF1* gene is located on chromosome 7p12.2 band and consists of 8 exons, encoding transcription factor IKAROS, which plays a key regulatory role in lymphocyte production (Rebollo & Schmitt, 2003). IKAROS contains six zinc finger structures, four of which are located in DNA binding domains encoded by exons 4 to 6 and are essential for maintaining IKAROS tumor suppressor function. The remaining 2 zinc fingers are encoded by exon 8 and mediate IKAROS as homologous dimerization or heterodimerization with other transcription factors of the family, such as AIOLOS and spirochetes (Stanulla et al., 2020). The presence of *IKZF1* deletion was associated with older age at diagnosis, higher white blood cell count, and higher minimal residual disease (MRD) levels after induction and consolidation (Mullighan et al., 2009; Waanders et al., 2011; Asai et al., 2013; Dorge et al., 2013; Palmi et al., 2013; Volejnikova et al., 2013; Yamashita et al., 2013; Vrooman et al., 2018; Yeoh et al., 2018). However, the distribution of *IKZF1* mutation in Chinese children with B-cell ALL (B-ALL) has been relatively poorly studied.

Here, we systematically analyzed the clinical and genetic characteristics of Chinese B-ALL children with *IKZF1* mutation in our single center. These data may provide evidence for risk stratification and individualized treatment for B-ALL.

## Methods

### Patients

A retrospective analysis was performed on 200 newly diagnosed patients with B-ALL aged 0–16 years who were admitted to the Children Hospital of Zhejiang University School of Medicine from 1 October 2017 to 31 August 2020. The diagnosis of B-ALL was based on the 2016 World Health Organization (WHO) classification criteria for hematopoietic and lymphoid tissue tumors (Arber et al., 2016). All patients were confirmed by comprehensive diagnosis of cytomorphology, immunology, cytogenetics and molecular biology, and complete medical history could be traced. Exclusion criteria: untraceable biological samples; unable to obtain necessary biological information; acute promyelocytic leukemia; other hematologic or non-hematologic tumors. The study was approved by the institutional review board of the Children’s Hospital of Zhejiang University Medical College and informed consents were obtained from patients and/or their legal guardians in accordance with the Declaration of Helsinki.

### Chromosome, Leukemia Fusion Gene and Flow Minimal Residual Disease Detection

Chromosomes were tested by Adicon Clinical Laboratory (Hangzhou, China). Leukemia fusion genes were sequenced by Kindstar Globalgene Technology (Wuhan, China). Flow cytometry (FCM) MRD were detected by the Children’s Hospital Leukemia Laboratory affiliated to the Children Hospital of Zhejiang University School of Medicine (Hangzhou, China). Hazard groups refer to CCLG-ALL-2008 scheme criteria (Brown et al., 2019; Brown et al., 2020). MRD detection for children examined by FCM: residual status of bone marrow tumor cells after induction (D_15_) and before consolidation (D_33_) treatment.

### Determination of *IKZF1* Large Segment Deletion by Multiplex Ligation-dependent Probe Amplification

Targeted copy number screening of the *IKZF1* gene was performed by multiplex ligation-dependent probe amplification (MLPA). The children’s mono-nuclear cells were retained at the initial diagnosis. DNA was extracted and analyzed using the SALSA MLPA KIT P335-B1 ALL-*IKZF1* probemix according to the manufacturer’s instructions. This SALSA contained a probe for each *IKZF1* exon. All MLPA reactions, including DNA denaturation, hybridization, ligation, and PCR, were carried out in a 96-well PCR thermocycler. The amplification products were quantified and identified by capillary electrophoresis. Normalization of the data was carried out by dividing the peak area of each probe by the average peak area of the control probes. This normalized peak pattern was divided by the average peak pattern of all the samples in the same experiment. The resulting values were 0–1 for every wild-type peak, 0.5 for heterozygous deletions and 1.5 for heterozygous duplications.

### Targeted Next-Generation Sequencing

DNA was extracted from whole bone marrow collected at diagnosis. Based on next-generation sequencing (NGS) of targeted capture, the mutation hotspots or entire coding region of 185 genes known to mutate frequently in hematological malignancies were sequenced (Supplementary Table S1). The following criteria were used to filter raw variant results: average effective sequencing depth on target per sample ≥1,000×; mapping quality ≥30; and base quality ≥30; variant allele frequency (VAF) ≥1% for SNVs and small InDels. Burrows-Wheeler alignment (BWA, version 0.7.12) was performed to align the trimmed reads. MarkDuplicates tool from Picard was used to mark the PCR duplicates. IndelRealigner and BaseRecalibrator from Genome Analysis Toolkit (GATK, version 3.8) were applied for realignment and recalibration of the BWA data, respectively. Variant calling, including SNVs and small InDels, was performed in Mutect2. ANNOVAR software was used to annotate all the variants including 1000G projects, COSMIC, SIFT, and PolyPhen.

### Statistics

Statistical analyses were carried out using R (version 3.5.2) or SPSS software (version 22.0). Mann-Whiney U test was used to compare the continuous variables. Chi-square test or Fisher’s exact test was used to compare the categorical variables. *p* < 0.05 was considered to indicate a statistically significant difference.

## Results

### Patient Characteristics

**FIGURE 1:**
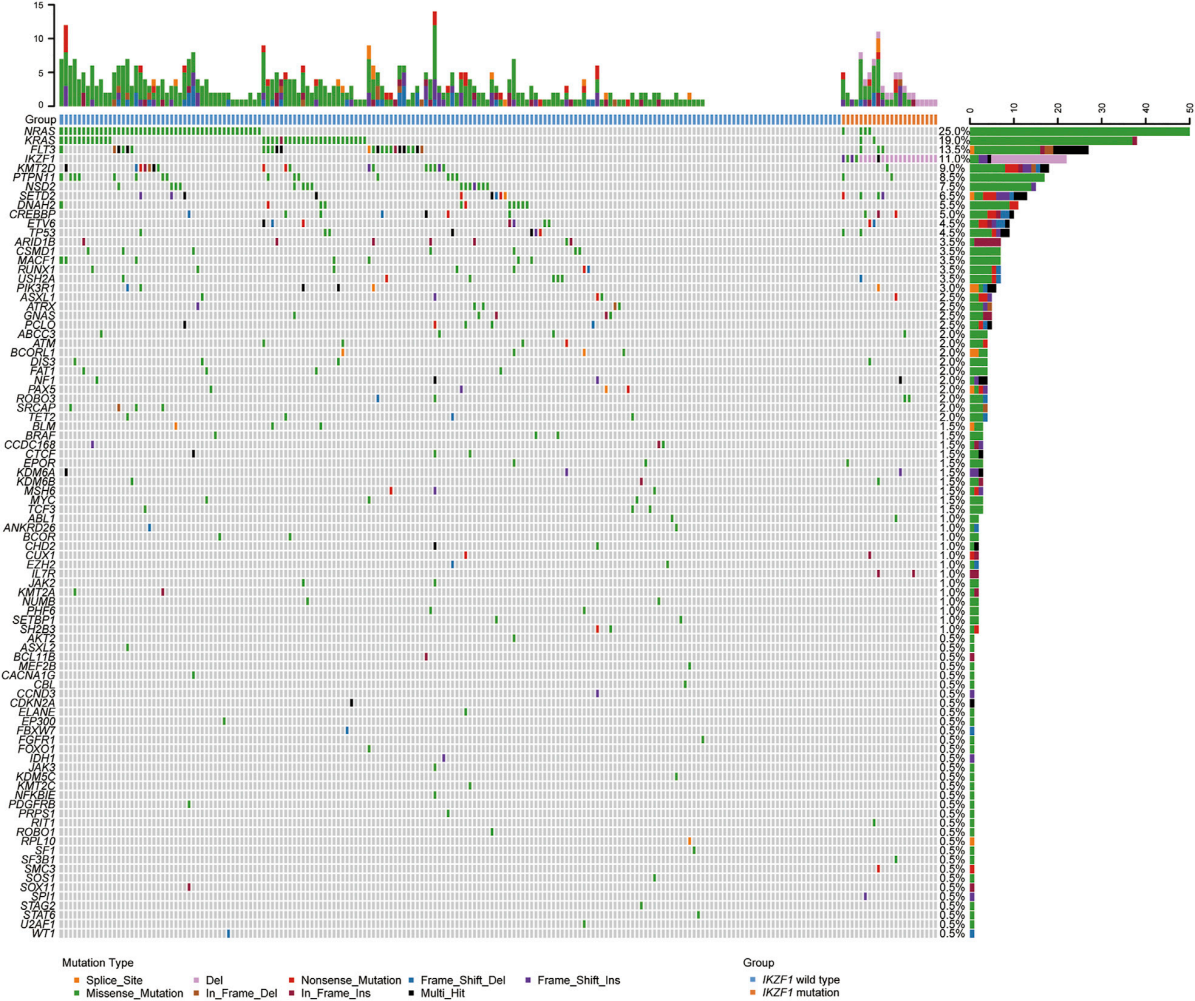
Overview of the gene mutations identified by targeted next-generation sequencing and multiplex ligation-dependent probe amplification in 200 B-ALL patients. Heatmap shows the specific mutations in each patient based on different gene mutation types, including large segment deletions, small insertions or deletions, and single nucleotide variations.

**FIGURE 2:**
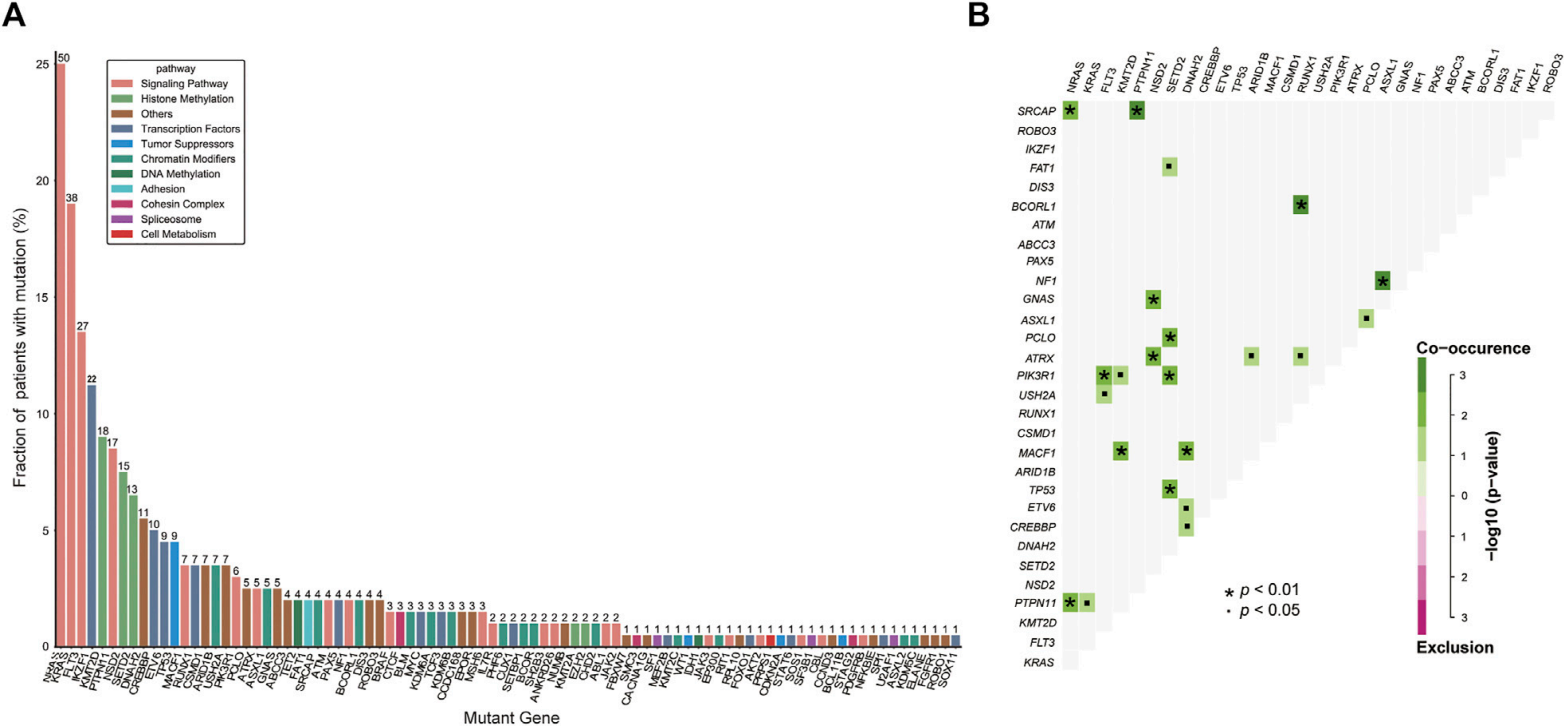
Genetic analyses in the whole cohort. **(A)** Histogram shows the frequency of gene mutations detected in the whole cohort according to the different functional groups assigned to each gene. **(B)** Diagram shows pairwise gene mutation correlations on the basis of the mutated genes detected in ≥2% patients. The odds ratio of the correlation is coded by different colors, and the significance level is marked by the symbol in each field.

A total of 200 B-ALL patients were enrolled in our study, including 106 males and 94 females, with a median age of 3.71 years (range, 0.05–16.25), as shown in Table 1. The median white blood cell (WBC) count, hemoglobin (Hb) concentration, and platelet (PLT) count was 6.49 × 10^9^/L, 81.00 g/L and 60.00 × 10^9^/L, respectively. Sixty-eight patients with a hyperdiploid chromosome karyotype (34.0%) were discovered in our cohort. On the 15th day of induction, 49 cases were with MRD >10^−3^ (24.9%). On the 33rd day of induction, 12 cases were with MRD >10^−4^ (6.0%). According to hazard groups from CCLG-ALL-2008 scheme criteria, 80 patents were assigned to the low-risk group (40%), 70 cases were in the intermediate risk group (35.0%) and 50 cases in the high risk group (25.0%). In 200 B-ALL patients, the overall rate of mutation prevalence was 82.0% (164/200) (Figure 1). A total of 88 mutated genes were detected, and the most common mutated gene was *NRAS* (25.0%), followed by *KRAS* (19.0%) and *FLT3* (13.5%) (Figure 2A). In total, 553 mutation sites were detected, and nonsynonymous SNV (65.0%) was the most common mutation type (Figure 1). Significant associations were discovered between mutated *SETD2* and mutations in *TP53*, *PCLO*, *PIK3R1* and *FAT1*, and between mutated *ASXL1* and *CHD2* and *NF1* mutations (Figure 2B).

**TABLE 1:** Clinical and genetic features in 200 B-ALL patients.

Characteristics	Total cohort, *N* = 200
Male, n (%)	106 (53.0%)
Female, n (%)	94 (47.0%)
Age, M (range) years	3.71 (0.05–16.25)
WBC, M (range) ×10^9^/L	6.49 (0.35–544.34)
Lymphocyte, M (range) ×10^9^/L	64.80 (0.06–97.10)
Neutrophil, M (range) ×10^9^/L	10.00 (0.00–80.20)
Hemoglobin, M (range) ×g/L	81.00 (27.00–129.00)
Platelet, M (range) ×10^9^/L	60.00 (3.00–483.00)
Insensitive to glucocorticoid, n (%)	30 (15.0%)
MRD >10^−3^ (the 15th day after treatment) n (%)	49 (24.9%)
MRD >10^−4^ (the 33rd day after treatment) n (%)	12 (6.0%)
Low risk, n (%)	80 (40.0%)
Intermediate risk, n (%)	70 (35.0%)
High risk, n (%)	50 (25.0%)

### Comparison of Clinical and Genetic Characteristics Between *IKZF1* Mutant and Wild-Type Patients

*IKZF1* mutations, including large segment deletions, small InDels and SNVs, were detected in 22 of 200 B-ALL children, with a positive mutation rate of 11.0%. The median WBC count in *IKZF1* mutant children was 19.38 × 10^9^/L, and was about 4 times higher than that in *IKZF1* wild-type children (*p* = 0.002). Both the median hemoglobin levels and platelet counts were not significantly different between *IKZF1* mutant and wild-type patients. More than half of cases with *IKZF1* mutation were not sensitive to glucocorticoid induction, and the proportion was more than 5 times higher than that of wild-type cases (63.6% vs. 9.0%, *p* < 0.001). On the 15th day of induction, 10 *IKZF1* mutant cases were MRD >10^−3^ by FCM, while 39 *IKZF1* wild-type cases were MRD >10^−3^ (45.5% vs. 22.3%, *p* = 0.018) (Table 2).

**TABLE 2:** Comparison of clinical and genetic features between *IKZF1* mutant and wild-type patients.

Characteristics	*IKZF1* mutant (*n* = 22)	*IKZF1* wild-type (*n* = 178)	*p-value*
Male, n (%)	14 (63.6%)	92 (51.7%)	0.280
Female, n (%)	8 (36.4%)	86 (48.3%)	
Age, M (range) years	5.90 (0.90–13.40)	3.50 (0.05–16.25)	0.700
WBC, M (range) ×10^9^/L	19.38 (3.08–544.34)	5.80 (0.35–515.00)	0.002
Lymphocyte, M (range) ×10^9^/L	58.50 (8.00–83.90)	65.70 (0.06–97.10)	0.190
Neutrophil, M (range) ×10^9^/L	6.00 (2.00–26.00)	10 (0.00–80.20)	0.170
Hemoglobin, M (range) ×g/L	84.00 (32.00–113.00)	81 (27.00–129.00)	0.940
Platelet, M (range) ×10^9^/L	43.50 (3.00–167.00)	62.00 (3.00–483.00)	0.065
Insensitive to glucocorticoid, n (%)	14 (63.6%)	16 (9.0%)	<0.001
MRD >10^−3^, n (%) (the 15th day after treatment)	10 (45.5%)	39 (22.3%)	0.018
MRD >10^−4^, n (%) (the 33rd day after treatment)	2 (9.1%)	10 (5.6%)	0.830

**FIGURE 3:**
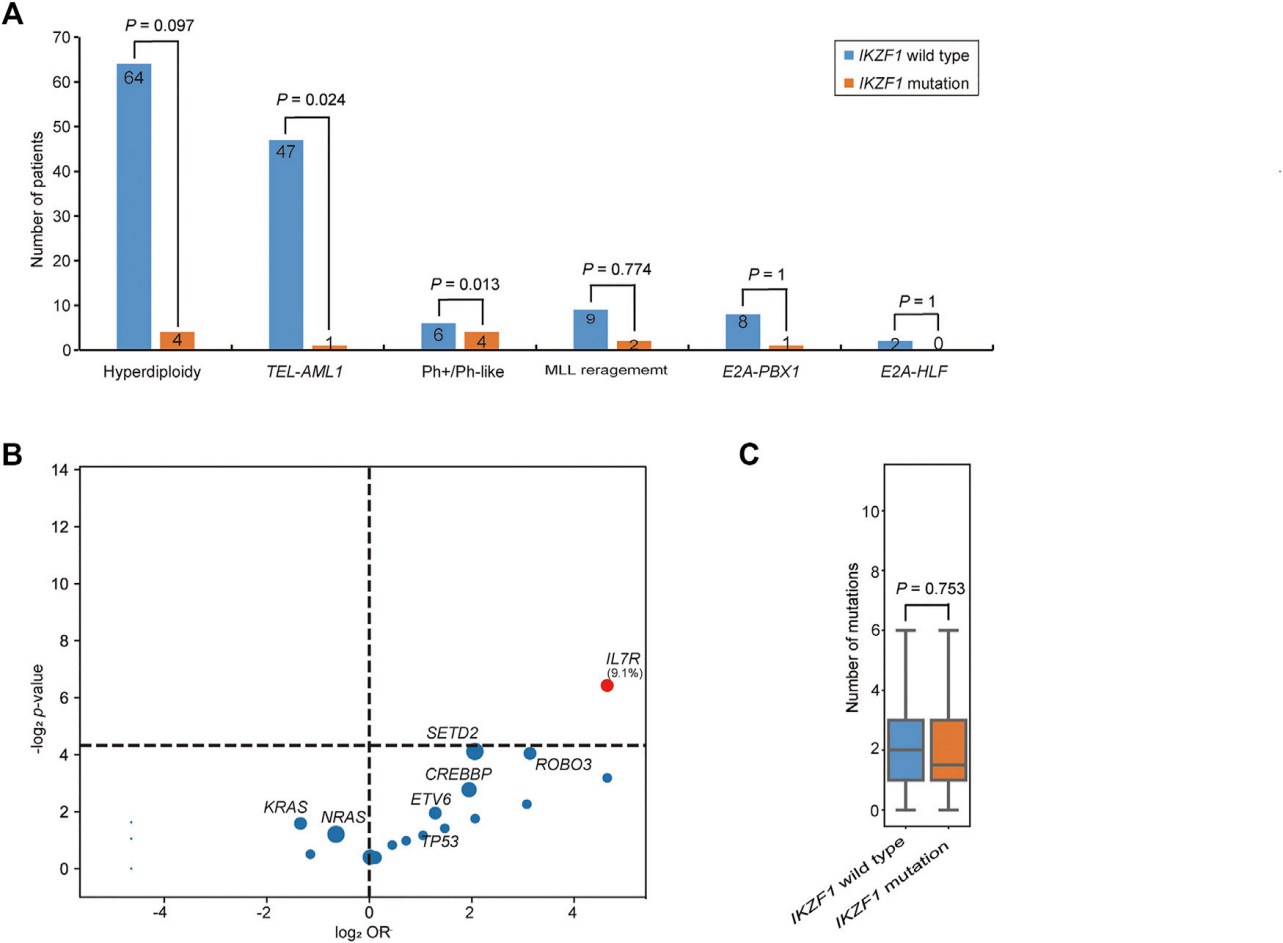
Comparison of genetic characteristics between *IKZF1* mutant and wild-type patients. **(A)** Bar chart shows the associations between *IKZF1* mutations and different cytogenetics or genetic aberrations. **(B)** Volcano plot shows the distribution of genetic characteristics according to *IKZF1* mutant and wild-type patients. The x axis indicates the magnitude of association (log_2_ odds ratio), and the y axis represents the −log_2_ *p* value. Each circle shows a mutated gene and the size of each circle represents the frequency of the mutated gene. **(C)** Box plot shows the comparison of the number of mutations between *IKZF1* mutant and wild-type patients.

The incidence of *IKZF1* mutation was shown in Figure 3A. 36.0% of *IKZF1* wild-type cases carried a hyperdiploid chromosome karyotype, while only 18.2% of *IKZF1* mutant cases were with hyperdiploid (*p* = 0.097). 97.9% of *TEL*-*AML1* positive B-ALL children had the wild-type *IKZF1*, and only one case had abnormal *IKZF1* (2.1%). For 11 MLL rearrangement positive B-ALL cases, *IKZF1* mutations were detected in 2 cases, and wild-type *IKZF1* was in 9 cases (18.2% vs. 81.8%). *IKZF1* mutation was not detected in the *E2A-HLF* subgroup (0%). Of the 9 *E2A*-*PBX1* positive cases, only one case had abnormal *IKZF1*, while of the 10 Ph+/Ph-like cases, 4 (40.0%) had *IKZF1* mutations. Compared with *IKZF1* wild-type cases, the interleukin (IL)-7 receptor (IL7R) gene mutation only occurred in *IKZF1* mutant cases, and the difference was statistically significant (9.1% vs. 0.0%, *p* = 0.012) (Figure 3B). *SETD2* and *ROBO3* mutations were found in 18.2% and 9.1% of *IKZF1* mutant cases, respectively, which seemed higher than that in wild-type cases (18.2% vs. 5.1%; 9.1% vs. 1.1%, respectively), but no statistical difference was discovered. Furthermore, based on the analysis of the number of mutated genes, there was no significant difference between *IKZF1* mutant and wild-type patients (*p* = 0.753) (Figure 3C).

### Association Analysis of Genetic Mutations in Patients With *IKZF1* Mutation

**FIGURE 4:**
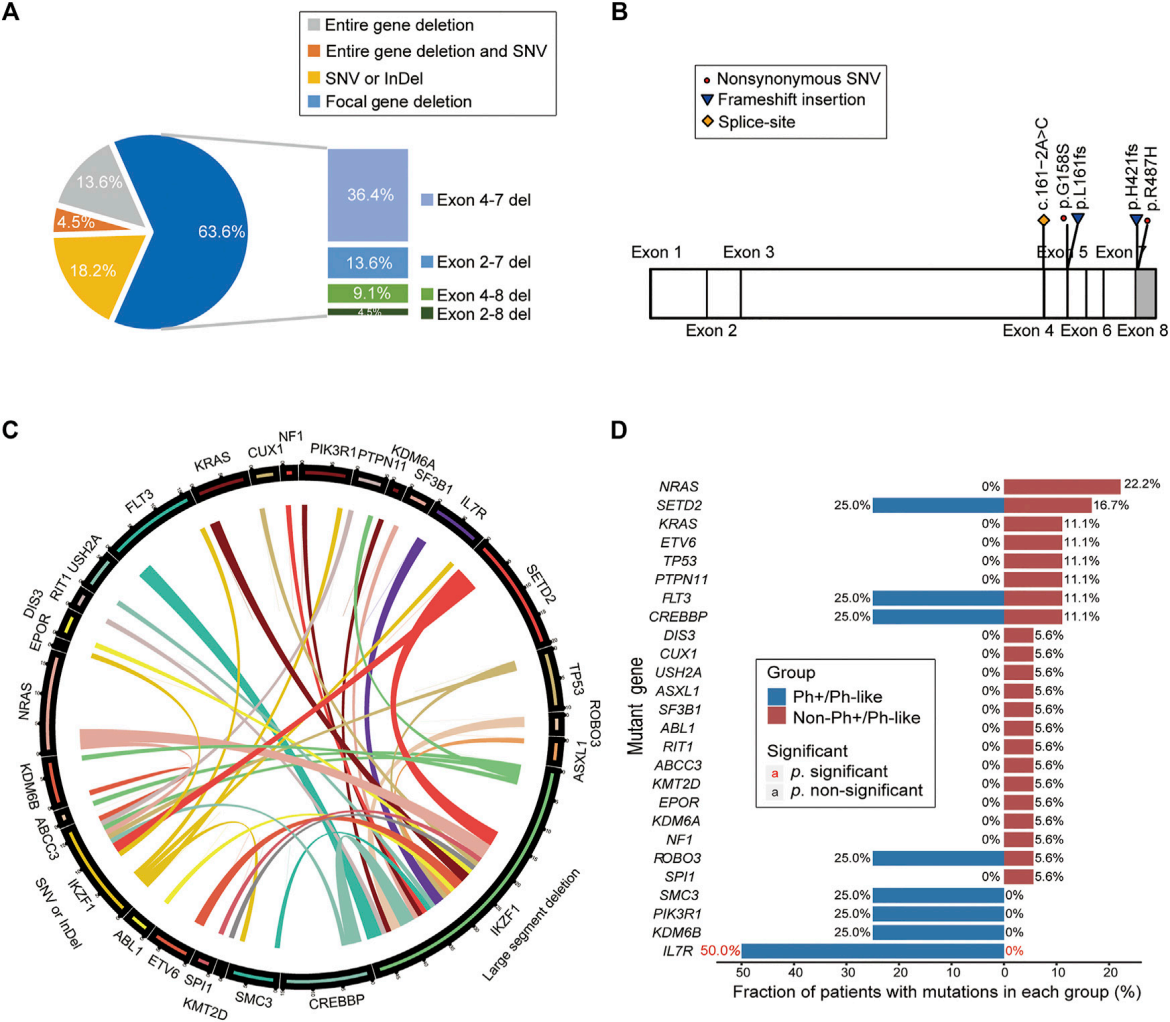
Analysis of gene mutation characteristics in patients with *IKZF1* mutation. **(A)** Pie chart shows specific types of *IKZF1* mutations, including the entire gene deletions, focal gene deletions, single nucleotide variations, and small insertions or deletions. **(B)** Schematic representation of the mutations detected in the *IKZF1* gene, only for single nucleotide variations, and small insertions or deletions. **(C)** Circos plot shows all the genetic mutations in the *IKZF1* mutation cohort, corresponding to the relative frequency and pairwise co-occurrence of gene mutations. The length of the arc indicates the frequency of mutations in the first gene, and the width of the ribbon represents the percentage of patients carrying the second gene mutation. **(D)** Comparison of the mutational genotypes of Ph+/Ph-like positive and Ph+/Ph-like negative B-ALL with *IKZF1* mutation. Percentage frequencies in each group are depicted.

Among 22 *IKZF1* mutant cases, 17 cases carried only *IKZF1* large segment deletion, 4 cases had SNV or small InDel mutations in the *IKZF1* gene, and 1 case had both *IKZF1* large segment deletion and SNV mutation**.** For large segment deletion, 4 (18.2%) cases involved exon 1–8 deletion of the entire gene, while 14 (63.6%) cases involved focal gene deletion, including exon 4–7 deletion in 8 cases (36.4%), exon 2–7 deletion in 3 cases (13.6%), exon 4–8 deletion in 2 cases (9.1%), and exon 2–8 deletion in one case (4.5%) (Figure 4A). For *IKZF1* SNV and small InDel mutations, the main types were frameshift and missense mutations, two of which were located in the zinc finger structure of exon 4–7, including *IKZF1* G158S and L161fs (Figure 4B). Based on the analysis of two different *IKZF1* mutation types, including large segment deletion, and SNV or small InDel, the correlations between paired genes were revealed (Figure 4C).

According to the distribution of mutations in 22 *IKZF1* mutant patients, 26 mutant genes were discovered, and *NRAS*, *SETD2*, *FLT3*, *CREBBP* were common detected genes (Supplementary Figure S1A). The cluster analysis based on gene function pathways showed that the mutant genes were mainly related to signaling pathway (40.5%) and transcription factor (16.7%) (Supplementary Figure S1B). The *IL7R* mutation accounted for 50.0% of B-ALL cases with Ph+/Ph-like combined with *IKZF1* abnormalities (50.0% vs. 0%, *p* = 0.03) (Figure 4D).

## Discussion

In this study, we systematically identified clinical and genetic characteristics of Chinese B-ALL children with *IKZF1* mutation. *IKZF1* mutation has been a hot topic in the field of leukemia since Mullighan *et al.* firstly reported in ALL patients in 2008 that single allele focal deletion affects its coding region (Mullighan et al., 2008). *IKZF1* mutation will lead to the obstruction of lymphocyte differentiation and development, resulting in leukemia. Based on MLPA to determine the partial or complete *IKZF1* large segment deletions, our study found *IKZF1* large segment deletion frequency was 9.0%, which was consistent with the result that Asai *et al.* reported 19 of 202 (9.4%) patients were carrying *IKZF1* large segment deletions (Asai et al., 2013). However, other studies observed differences in *IKZF1* large segment deletion frequency: 12% in German patients, 20.6% in Mexico patients, 16% in Swedish patients, and 28.6% in American patients. *IKZF1* SNV and small InDel mutation in our study was 2.5%, almost close to the proportion reported in the literature (<1%) (Mullighan et al., 2008; Dorge et al., 2013; Ofverholm et al., 2013; Ayon-Perez et al., 2019; Stanulla et al., 2020).

Previous studies showed that the different type of *IKZF1* mutations, including large segment deletion, small InDel and SNV, produced different molecular results. Deficiencies from these sites, such as the entire gene (including exons 1–8), or focal gene (including exons 2 and/or 8), or untranscriptional regulatory regions (including exon 1), can lead to *IKZF1* hypofunction (Iacobucci & Mullighan, 2017). The deletion of exons 4–7, lacking the ability to bind DNA, was a negative domain and thus led to leukemia (Mullighan et al., 2008; Iacobucci et al., 2009; Chiaretti et al., 2016), which was the most common deletion pattern of *IKZF1* in our cohort (36%), consistent with results in Germany, Japan, Sweden and the US and different from results in Mexico, where the deletion of exon 1 (85%) occurred most frequently (Ayon-Perez et al., 2019). Although SNV or small InDel mutations of *IKZF1* were infrequent and present, their molecular consequences could be either haploid insufficiency or dominant negative effects, as with deletions. Given this, the molecular effects of these types of mutations can be further judged by gene expression. Recent research discovered that *IKZF1* missense mutation (p.N159Y) affect the DNA binding domain and validated by gene expression profile (Li et al., 2018; Gu et al., 2019). Five *IKZF1* mutations, including SNV or small InDel mutations, were also detected in our study, whose final molecular effects need to be further clarified by transcriptome sequencing.

However, *IKZF1* is controversial as an independent risk factor for patient prognostic stratification. Some studies suggested *IKZF1* large segment deletion was closely related to the high recurrence and low survival of pediatric B-ALL (Kuiper et al., 2010; Yang et al., 2011; Buitenkamp et al., 2012). Boer *et al.* showed that any kind of *IKZF1* large segment deletion increased risk compared to patients with wild-type *IKZF1*, based on their high WBC count >50,000/µl (Boer et al., 2016). Indeed, we observed this phenomenon for 22 patients with *IKZF1* mutations in this study, who had higher levels of leukocytes at the time of initial diagnosis, insensitivity to glucocorticoid, and higher levels of MRD on day 15th of induction remission.

We also found that the partner mutant genes associated with the *IKZF1* mutations are closely related to the signaling pathway and transcription factor function (*NRAS*, *SETD2*, *FLT3* and *CREBBP*). In particular, the mutation in *IL7R* was only found in *IKZF1* mutation cases, suggesting the *IL7R* mutation may be synergistic with the *IKZF1* mutation and participate in the occurrence of the B-ALL. In our study, these two patients with *IL7R* mutation were insensitive to glucocorticoid therapy. Several research suggested that *IL7R* functional acquired mutations made *IL7R* highly expressed, and *IKZF1* deletion deprived the IKAROS of its inhibitory effect on the promoter region. *IKZF1* and *IL7R* synergistically activated downstream JAK/STAT5 and PI3K/Akt/mTOR signaling pathways to promote leukemia (Ge et al., 2016). Thomas et al. (2021) showed that *IL7R* mutation led to B-ALL alone in a mouse model and *IKZF1* mutation contributed to the process of leukemogenesis.

Our study has several limitations. First of all, due to the lack of follow-up data, the long-term prognostic value of *IKZF1* mutation remains to be explored. Secondly, this study is a single-center result, which may not fully reflect the distribution of clinical and genetic characteristics in the Chinese population. Therefore, large-scale multi-center studies and long-term follow-up should be included in the future.

Inconclusion, our research showed clinical and genetic characteristics of *IKZF1* mutation in Chinese Children with B-ALL. This study reveals the association between genetic mutations and clinical features. These investigations might contribute to molecular classification, risk stratification and prognosis evaluation, and provide new ideas for targeted therapy.

## Data Availability

The data that support the findings of this study have been deposited into CNGB Sequence Archive (CNSA) of China National GeneBank DataBase (CNGBdb) with accession number CNP0002707 https://db.cngb.org/search/project/CNP0002707.
