# Supplementary material for: Clinical and Genetic Characteristics of IKZF1 Mutation in Chinese Children With B-Cell Acute Lymphoblastic Leukemia
Source: Front Genet. 2022 Mar 28;13:822832. doi: 10.3389/fgene.2022.822832 (PMC9000999; doi:10.3389/fgene.2022.822832)
Supplement: Supplementary file 1 [file Table1.DOCX]

**Supplementary Table 1. Genetic mutation list in 185-gene panel**

| ABCB1 | ABCC3 | ABCG2 | ABL1 | ADSL | AKT2 | ALK | ANKRD26 |
| --- | --- | --- | --- | --- | --- | --- | --- |
| ARID1B | ASNS | ASXL1 | ASXL2 | ATM | ATRX | B2M | BCL11B |
| BCL2 | BCL6 | BCOR | BCORL1 | BLM | BMP7 | BRAF | CACNA1G |
| CBL | CCDC168 | CCND3 | CD79A | CDA | CDKN1B | CDKN2A | CEBPA |
| CECR2 | CEP72 | CHD2 | CPA2 | CREBBP | CRLF2 | CSF3R | CSMD1 |
| CTCF | CTLA4 | CTNNB1 | CUX1 | CYP2B6 | CYP2C19 | CYP2C8 | CYP3A4 |
| CYP3A5 | CYPA | DARS | DDX41 | DHX15 | DHX30 | DIS3 | DNAH2 |
| DNM2 | DNMT3A | DOK5 | DORSHA | DRB1 | DYNC2H | ELANE | EP300 |
| EPOR | ERCC1 | ETNK1 | ETV6 | EZH2 | FAM46C | FAT1 | FBXW7 |
| FGFR1 | FLT3 | FOXO1 | GART | GATA1 | GATA2 | GATA3 | GFI1 |
| GNAS | GRIA1 | GSTM1 | GSTP1 | HAX1 | ID3 | IDH1 | IDH2 |
| IKZF1 | IL7R | IMPDH2 | ITPA | JAK1 | JAK2 | JAK3 | KDM5C |
| KDM6A | KDM6B | KIT | KMT2A | KMT2C | KMT2D | KRAS | LINC00251 |
| MACF1 | MAP2K1 | MED12 | MEF2B | MPL | MSH6 | MTRR | MYC |
| MYD88 | NF1 | NFKBIE | NOTCH1 | NPM1 | NR3C1 | NRAS | NSD2 |
| NT5C2 | NUDT15 | NUMB | PAX5 | PCLO | PDGFRA | PDGFRB | PHF6 |
| PIGA | PIK3R1 | PLCG2 | PNPLA3 | PPM1D | PRKDC | PRPF8 | PRPS1 |
| PTEN | PTPN11 | RIT1 | ROBO1 | ROBO3 | RPL10 | RRM1 | RRM2 |
| RUNX1 | SETBP1 | SETD2 | SF1 | SF3B1 | SH2B3 | SLCO1A2 | SLCO1B1 |
| SMC1A | SMC3 | SOS1 | SOX11 | SPI1 | SRCAP | SRGAP1 | SRGAP2 |
| SRGAP3 | SRP72 | SRSF2 | STAG2 | STAT3 | STAT5B | STAT6 | TCF3 |
| TERT | TET2 | TNF | TNFAIP3 | TNFRSF14 | TP53 | TPMT | TRIM24 |
| U2AF1 | UGT1A1 | UGT1A8 | USH2A | USP7 | VEGFC | WT1 | XRCC5 |
| ZRSR2 |  |  |  |  |  |  |  |
