# Supplementary figures and images for: Clinical and Genetic Characteristics of IKZF1 Mutation in Chinese Children With B-Cell Acute Lymphoblastic Leukemia
Source: Front Genet. 2022 Mar 28;13:822832. doi: 10.3389/fgene.2022.822832 (PMC9000999; doi:10.3389/fgene.2022.822832)

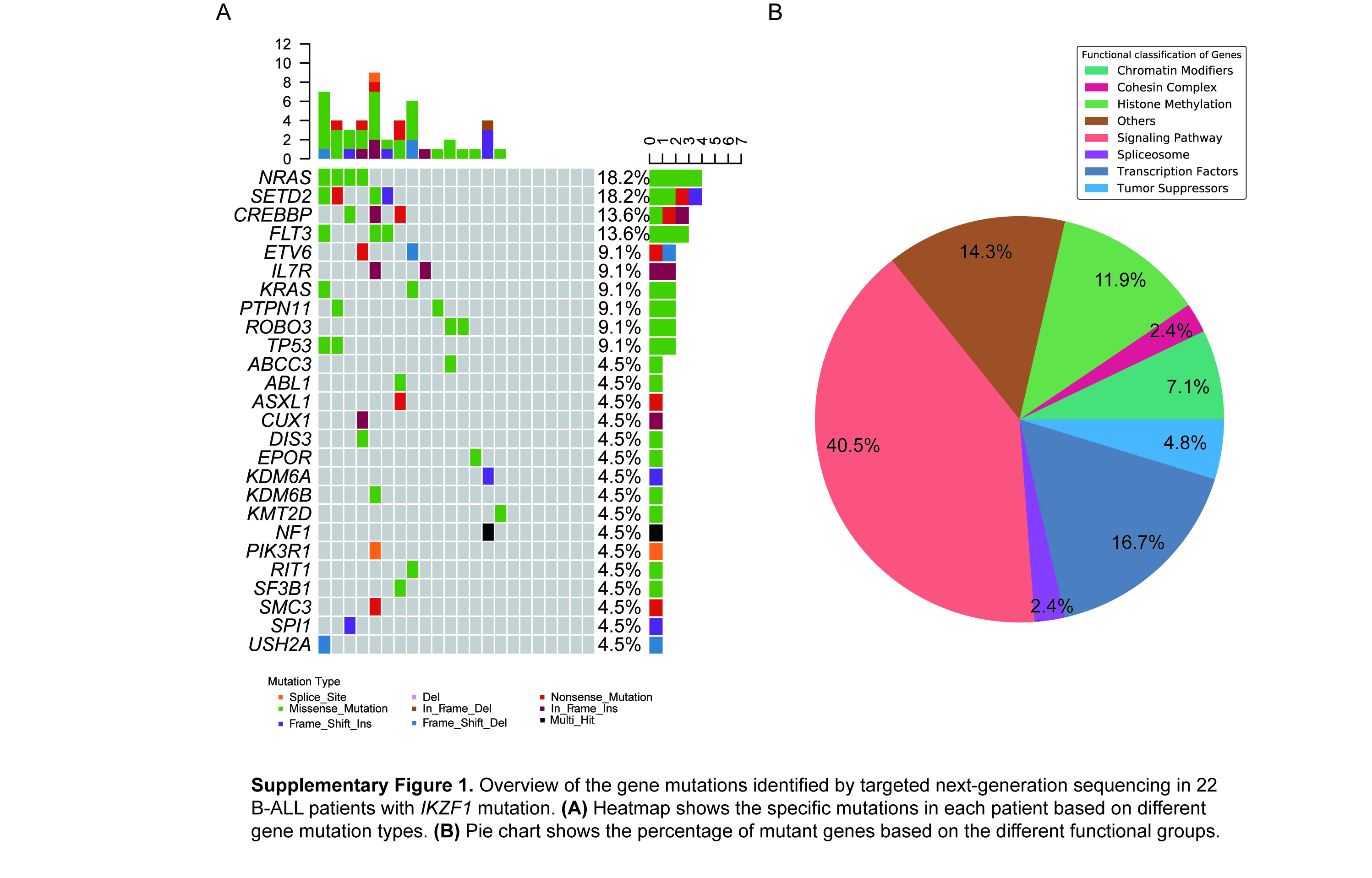

Supplement: Supplementary file 2 [file Image1.TIF]
